# Behavioral Indices of Neuropsychological Processing Implicated in Moral Domain Reasoning amongst Children and Adolescents

**DOI:** 10.3390/brainsci9120331

**Published:** 2019-11-20

**Authors:** Simona C. S. Caravita, Lisa Astrologo, Giulia Biancardi, Alessandro Antonietti

**Affiliations:** 1 Department of Psychology, Catholic University of the Sacred Heart, 20123 Milano, Italy; biancardi.giulia@gmail.com (G.B.); alessandro.antonietti@unicatt.it (A.A.); 2 Department of Clinical Psychology, Concordia University, Montreal, QC H3G 1M8, Canada; lisa.astrologo@live.ca

**Keywords:** moral domains, moral reasoning, age-related differences, behavioral indices, socio-conventionality, children, adolescents

## Abstract

Moral domain theory posits that moral knowledge is organized in separate domains related to moral and socio-conventional rules, with the latter being reliant on a statement made by authority. Domains may be contingent on different neuropsychological processing that may vary with age. Behavioral indices were measured in three age groups, to detect differences in the neuropsychological processing allegedly involved in the evaluation of rule transgressions in different domains. Acceptance of the transgressions was also investigated. Twenty-four children, 32 early adolescents, and 31 adolescents judged acceptability of rule transgressions when an authority figure allowed the transgression. Across age, moral-rule transgressions were less accepted and took significantly longer to be evaluated. In evaluating moral rule scenarios, children had the longest reaction times. Older adolescents took the least amount of time evaluating socio-conventional rule scenarios. Results suggest differences in the neuropsychological processing underlying decision making for moral and socio-conventional domains and that rule comprehension and distinction amongst domains increase by age.

## 1. Introduction

According to Turiel’s [1] theory in *The Development of Social Knowledge*, the concept of moral decision making depends heavily on social understanding, which begins to be accumulated in early childhood. Throughout development, the knowledge acquired through the environment produces distinct categories of reasoning that are heavily implicated by social interactions and cultural connotations, which are later organized and contextualized by the child to be used in future situations and social interactions. In the present study, this framework was used to explore age-related differences of moral development through behavioral indices in children, early adolescents, and adolescents. We hypothesized that children, early adolescents, and adolescents would evaluate moral scenarios in different manners, depending on the type of rule and the related moral knowledge involved in the scenarios. Similarly, we expected participants to differ in the time it would take to evaluate transgressions with regard to both age of participant and type of scenario, thus expressing possible differences in the organization of the knowledge.

Each separate domain is compliant to a set of rules. According to Turiel’s theory [1] there are two sets of rules in moral decision making: (1) moral rules and (2) socio-conventional rules. Moral rules are set to preserve others’ well-being (e.g., rules that prohibit stealing) whereas the socio-conventional rules aim to preserve social order and are typically implemented by figures of authority (e.g., dress regulations at schools). These rules are useful in examining the boundaries of morality in individuals and to determine the specific contexts and domains relevant in assessing moral transgressions.

There are three judgment criteria implicated in determining the difference between moral and social-conventional violations amongst individuals [2]. To begin, unlike moral rules, socio-conventional rules are dependent on societal standards and authorities’ statements [3]. Next, moral rules are typically universal, and transgressions of moral rules will be considered unacceptable in various contexts [4,5]. Finally, and most importantly, moral rules are thought to be unchangeable, whereas socio-conventional rules are typically implemented by an authority figure or based on societal standards and will vary depending on the context [6]. Thus, it can be argued that different and distinguished domains of knowledge organization are involved in the process of assessing moral and socio-conventional transgressions [4,6]. The critical difference between moral and socio-conventional rules is the role that an authority figure can play in establishing the acceptability of a transgression. Authority figures do not influence the value of moral rules, but they influence socio-conventional rules, as they are context-dependent and will vary depending on the norms of the time [4]. This study is interested in exploring this concept further by analyzing the flexibility of socio-conventional rules by manipulating authority statements.

Current evidence suggests that there are further differences when evaluating the acceptability of moral and socio-conventional transgressions [4]. Moral-rule transgressions are typically universally wrong, whereas socio-conventional transgressions are typically evaluated as being more acceptable [4]. This may be due to the variation of contexts that are specific to the socio-conventional rules, which are typically based on an authority figure and may not apply to all situations and contexts, whereas moral rules are universal in nature [7]. Thus, moral rule transgressions are deemed more serious than socio-conventional rule transgressions [4,6]. Accordingly, the statement of an authority figure seems to play a critical role in determining the acceptability of a transgression within a moral or socio-conventional context. 

To our knowledge, our study is one of the first to prioritize socio-conventionality in moral development. The authority figure (the school principal in our study) will be the only figure either allowing a transgression or enforcing a rule. In other words, participants would be evaluating the socio-conventional transgressions based purely on the statement of the authority figure. This characteristic of the scenarios we used will allow for better analysis in determining the context of accepting transgressions of different types of rules. 

### 1.1. Neuropsychological Indices in Moral Development

The moral domain theory focuses on the different domains implicated in moral reasoning. This theory does not, however, lend itself to understanding the neuropsychological processes behind moral decision making [2]. While the literature is sparse, current research demonstrates evidence (e.g., [8,9,10]) that different brain regions are activated when being asked to evaluate different types of moral situations. Furthermore, adolescents with traumatic brain injury tend to have less mature responses to moral dilemmatic scenarios than typically developing individuals, which suggests the implications of specific brain regions for moral development [11]. 

Areas of the brain that are typically associated with emotional processing (i.e., posterior cingulate gyrus and the bilateral angular gyrus) were significantly more active when processing intuitively “up close and personal” and more emotional scenarios (personal dilemmas) than more intuitively impersonal and less emotional scenarios (impersonal dilemmas) [8]. Similarly, brain areas usually associated with working memory were more active when presented with impersonal dilemmas [8].

The responses for personal problems, which are usually based on rights and duties, were found to be emotionally charged, whereas impersonal moral dilemmas were more often based on conscious and controlled thinking, which required utilitarian judgements, such as evaluating cost–benefit balance [8]. Thus, the amount that an individual is implicated within the decision making of a moral dilemmatic situation will influence the neuropsychological processes involved in evaluating the situation [8]. Accordingly, Han and colleagues [10] found that, in comparison to moral impersonal dilemmas, moral personal dilemmas evoked longer response times, as well as elicited activation in different brain areas.

While these neuropsychological processes are rarely explored in the moral domain theory (to our knowledge, only one study [12] investigated evaluations of moral and socio-conventional domains in traumatic brain injury patients), these findings provide an interesting point of reference when examining the brain functioning’s behind moral processing. If different knowledge organizations are implicated in evaluating different types of moral scenarios, in which different types of rules (moral vs. socio-conventional) are broken, the type of scenario may predict the amount of time it takes to evaluate the acceptability of the transgression. 

Current research examining behavioral indices in the moral domain framework has determined that the amount of time it takes to evaluate the acceptability of a moral transgression differs with age [2]. However, to our knowledge, the current literature has yet to examine whether these neuropsychological processes explain the time differences in evaluating transgressions. Our study aims to further the understanding of behavioral indices implicated in evaluating moral and socio-conventional scenarios in different age groups.

### 1.2. Age-Related Differences in Moral Domains

Age plays a role in the evaluation of situations related to moral and socio-conventional rules [2,5]. Age-related differences were discovered when participants were making normative judgements, that is, children accepted less the transgressions of all types of rules than adolescents [4]. Children tend to respond differently than adolescents to moral decision making, such that children were less likely to attribute positive emotions to a victimizer in a moral transgression than adolescents and adults [13].

Furthermore, moral evaluation tends to change by age, in that adolescents are more likely to integrate the moral criteria in evaluating social situations and prioritize the moral rule when evaluating a transgression [4]. Similarly, socio-conventional transgressions tend to become more acceptable with age [4]. Thus, it could potentially be argued that the flexibility of socio-conventional rules, such that they are dependent on context and authority figure, is a concept that becomes more understood as development progresses.

In addition, before eight years of age, children do not characteristically expect negative emotions as a consequence to a moral transgression [14]. Similarly, adolescents tend to be more heavily influenced by self-evaluative emotions, whereas older individuals seem to focus on outcome-oriented emotions [15]. Situations involving evaluation of moral rules, in which social behavior is judged in accordance to rules, principles, and one’s own interests, are suggested to require more complex thinking, which may explain the age-related differences in responses to moral transgressions [16]. 

Most aforementioned research examined the experimental manipulation of scenarios to determine the mechanisms behind evaluating social situations. Current literature has begun to examine behavioral indices of decision making (e.g., reaction time) [2]. Lahat and colleagues [2] measured, beside the event-related potentials, the reaction times of 24 adolescents (*M_age_* = 13 years) and young adults (*M_age_* = 20 years) whom moral, socio-conventional, and neutral scenarios were dictated. Regardless of the age, moral scenarios were resolved faster than socio-conventional scenarios across all participants, when a rule to follow was established. 

To our knowledge, the literature is quite sparse in regard to examining behavioral indices, specifically reaction time, of youth, when evaluating transgressions across the moral domain theory. Only a few studies [2,17] examined the behavioral indices, that is, reaction time, of evaluating moral and socio-cultural transgressions. Our study aimed to fill the gap in the literature in two areas. First, our study looked at the behavioral indices across three different age groups and across the longest period of development that we are aware of in the literature. Moreover, these behavioral indices will allow us make inferences on the neuropsychological mechanisms implicated in evaluating moral and socio-conventional rule transgressions. In addition, the socio-conventional rule situations we used are novel. Specifically, the socio-conventional rules are dependent on an authority figure’s rules and independent of moral-rule-regulated influences. Put another way, we believe we have disentangled moral rules from our socio-conventional rules and this will allow for better distinguished scenarios and a better understanding of the contexts critical to evaluating moral situations across development.

### 1.3. Present Study 

The first goal of the study was to analyze reaction times and responses in three different types of scenarios: (1) moral rule scenarios, (2) socio-conventional rule scenarios, and (3) neutral scenarios. The neutral scenarios contained impartial situations and were used as a comparison baseline for the study. Participants were of three age groups: children (aged 9–11 years), early adolescents (aged 12–14 years), and adolescents (aged 15–17 years). Participants were asked to evaluate the appropriateness of a transgression when it would be allowed by an authority figure (i.e., the school principal). The literature posits that only socio-conventional scenarios should be dependent on authority [3]. Thus, it was expected that participants would react differently and take different amounts of time when assessing transgressions in moral and socio-conventional contexts (as contrasted with the neutral ones) allowed by authority. Differences in reaction time would suggest differences in the neuropsychological processing underlying evaluations of different rule domains, supporting the hypothesis that moral rule knowledge is organized in separate cognitive domains.

The second goal of the study was to explore the age-related differences amongst the three age groups in behavioral indices (i.e., reaction time) associated to the evaluation of rule breaking in different contexts. Knowing that evaluations of social scenarios change with age, we expect that children will take more time to process the moral rule scenarios than early adolescents and adolescents [17].

## 2. Methods 

### 2.1. Participants 

The sample of participants consisted of 87 students from primary, middle, and high schools from two cities in two of the largest cities in Northern Italy: Milan and Brescia. Participants were made up of 24 children in primary school (*M_age_* = 9.33 years; *SD* = 0.56, 12 females), 32 early-adolescents in middle school (*M_age_* = 12.53 years; *SD* = 0.62, 14 females), and 31 adolescents in high school (*M_age_* = 15.45 years; *SD* = 0.57). A majority of participants (93.1%) were born in Italy or of Italian lineage.

### 2.2. Measures

Moral, Socio-Conventional, and Neutral Scenarios. Specific scenarios were adapted [4] to evaluate the specific domains that are attributed to particular rules and acceptance of transgressions within moral and socio-conventional contexts. Each moral and socio-conventional scenario was composed of a short story, where either a moral or a socio-conventional rule was violated. In neutral scenarios in which participants had to decide about allowability of actions that were regulated by neither moral nor socio-conventional rules. All the scenarios were under the assumption of happening at school and this assumption was explained to participants before the administration sessions. Participants were given 21 scenarios of moral-rule transgressions (e.g., “(At your school…) … hurting others is forbidden. While you are playing with the ball at school during recess, you give your opponent a push to make a point”), 20 scenarios of socio-conventional rule transgressions (e.g., “(At your school…) … arriving late to lectures is forbidden. One morning at school, you decide to talk to a friend rather than make it to class on time when the bell rang”), and 20 neutral scenarios (e.g., “(At your school…) ….one morning in class, you decided to remove your sweater”). All items required the participant to assume the perspective of protagonist of the short story, i.e., the rule-breaker in moral and socio-conventional scenarios. In all scenarios, the transgressive action/neutral action was presented as allowed by the school principal, as the main school-context authority. The scenarios were equally divided into male and female characters within the short stories.

### 2.3. Procedure 

This experiment was part of a larger study that was carried out in collaboration with the IRCCS “Eugenio Medea” rehabilitation center. Complete ethical permission was obtained by the IRCCS “Eugenio Medea”, as well as the individual study included in this paper. Written and informed consent was obtained by all parents via a letter sent home, detailing the premise of the study as in accordance with the Declaration of Helsinki. In addition, all children gave verbal consent before participating in the study and were reminded that they could withdraw at any point.

The scenarios were administered at school, during school hours, in a single session per each participant. Each session lasted a maximum of 90 min. A trained research assistant supervised the administration. Before the administration of the scenarios started, a preliminary session took place, during which the research assistant explained to each participant that all the scenarios were under the assumptions (1) of happening at school and (2) that the principal of the school was allowing the actions described in the stories (i.e., all scenarios were under the pretense of an authority figure allowing the potential transgression in the model). Then, in the same preliminary session, the research assistant trained each participant to answer the scenarios by using three scenarios that were not included in the test sets of stories (e.g., preliminary scenario: “The waste must not be left on the ground. One day the bell rings and you leave the class without picking up some of your papers from the ground”).

In both the preliminary and test sessions, each scenario was audio-recorded and was dictated to the participant. Scenarios were administered by using the Precision Teaching Software [18]. This software also allowed children to view images while listening to the audio recordings of the scenarios. Two pictures were included: one of the school principal and one of the school itself. The picture of the school principal was used to remind participants of the rule and that the transgression was allowed by the authority figure.

Participants were asked to judge whether an action was either acceptable or unacceptable in moral, socio-conventional, and neutral context scenarios in which the authority figure consented to the transgression. Each scenario ended with the question, “Is it right to do so?” and was coded 1 if the transgression of the rule was accepted and 0 if the transgression was deemed unacceptable by the participant. The average of item scores for each type of scenario was computed. A higher average signifies a greater acceptance of transgressions. Moreover, reaction times in making the decision of accepting/not accepting the action were recorded by means of the Precision Teaching Software. Precision Teaching Software records reaction times with a temporal resolution of 1 ms (1/1000 s). 

Scenarios were dictated to all participants in order to facilitate measuring the exact time it took for children to decide whether to accept or not accept the transgression, without possible variations caused by the time it takes children/adolescents to read a written scenario. Timing started at the end of dictating each scenario. Furthermore, for children in early primary school, listening comprehension is more developed than reading comprehension, as it often predicts reading ability later on in life [19]. Thus, to ensure equal understanding across participants, scenarios were dictated to all participants.

Two versions of the measure were created. One version was used for children, and the other version was used for the early adolescents and adolescents. The two versions were equivalent in content, word number, and grammatical structure. The versions only differed in contextual factors within the scenario (i.e., specifics of a situation) in order to be more relatable for the groups (i.e., children versus adolescents). 

## 3. Results 

To begin, we computed the descriptive statistics of our measures and demographic criteria to understand the association of the latter with the former with the time it took for the participants to evaluate transgressive/neutral actions and whether or not the type of actions was accepted. In addition, these associations were explored through intercorrelation indices. These results are reported in Table 1 and Table 2.

Across all three age levels, the socio-conventional scenarios were solved in the least amount of time, followed by moral scenarios, and lastly neutral scenarios. Children took the longest, whereas adolescents took the least amount of time to determine the acceptability of transgressive/neutral actions for all types of scenarios. Across all three age levels neutral scenarios had the highest mean of accepted actions, followed by socio-conventional transgressions and lastly moral transgressions.

Gender was not related to the time it took for participants to evaluate all three types of actions. The age of participants was negatively associated with the amount time they took to determine the permissibility of an action across all age groups for moral and socio-conventional scenarios. The older the participant, the less time it took to determine the acceptability of a transgression of a moral and socio-conventional nature.

Furthermore, both age and gender were unrelated to the time participants spent to assess neutral actions. This is expected, given the lack of meaning to these scenarios, which were not anticipated to behave in any particular way across gender and age groups. 

The associations for acceptability of transgressive/neutral actions for all three types of scenarios with demographic variables from the whole sample are summarized in Table 1. Gender was unrelated to participants accepting all types of actions.

Age shared a statistically significant and positive association with the acceptability of socio-conventional transgressions. Put another way, the older the participants, the more likely they were to accept socio-conventional transgressions.

### 3.1. Scenario Reaction Times Across Age 

Next, guided by the associations, we ran analyses of variance, to determine how age and type of scenario predicted the time it took for participants to evaluate and their likelihood of accepting the actions. To begin, we added type of scenario as within subject factors and gender and age as between subjects’ factors in a 3 × 3 × 3 repeated measure ANOVA. It was noted that gender continued to be insignificantly associated (statistically) with the predictors and was subsequently removed from the model. Nevertheless, as the literature indicates that there is possible variation in moral reasoning by gender [4], gender was added as a covariate to control for its effect and to obtain purer estimates of the effects by the other factors. 

A 3 within subjects’ factor (socio-conventional × moral × neutral scenarios) by 3 between subjects’ factor (children × early adolescent × adolescent) ANCOVA (gender as covariate) was then conducted to determine the relationship between scenario reaction times across age. There were significant main effects of scenario type within subjects, *F* (4, 166) = 27.96 *p* < 0.05, Partial *η*^2^ = 0.252, and of age between subjects, *F* (1, 83) = 9.85, *p* < 0.05, Partial *η*^2^ = 0.192. The main differences to note are that socio-conventional scenarios took the least amount of time, with neutral scenarios taking the longest to be completed, and that reaction time decreased by age. There was also a significant interaction effect between the type of scenario and the age of participants, *F* (4, 166) = 5.88, *p* < 0.05, Partial *η*^2^ = 0.124. Thus, the amount of time participants took to evaluate a transgressive/neutral action is dependent on both the type of actions they are evaluating and their age. 

In order to interpret the interaction effect, follow-up analyses of variances were conducted for each age group. The time to evaluate the actions significantly differed by age groups across all types of scenarios (moral scenarios: *F* (2, 83) = 4.66, *p* < 0.01, Partial *η*^2^ = 0.144; socio-conventional scenarios: *F* (2, 83) = 6.69, *p* < 0.001, Partial *η^2^* = 0.195; neutral scenarios: *F* (2, 83) = 8.56, *p* < 0.001, Partial *η*^2^ = 0.236). Pairwise comparison analyses showed that children took significantly (*p* < 0.05) longer time to evaluate moral and neutral scenarios in comparison to the early adolescents and adolescents. In addition, adolescents, the oldest age group, took significantly less time to evaluate socio-conventional scenarios in comparison to children and early adolescents, but children and early adolescents did not significantly differ from each other (*means* and *SDs* are reported in Table 1). Furthermore, repeated-measure follow-up analyses were conducted to determine the effect of type of scenario on reaction time across participants. These analyses determined that the amount of time to evaluate a scenario significantly differed across all three types of scenarios (children: *F* (2, 44) = 10.43, *p* < 0.001, Partial *η*^2^ = 0.322; early adolescents: *F* (2, 60) = 5.84, *p* < 0.01, Partial *η*^2^ = 0.163; adolescents: *F* (2, 58) = 18.79, *p* < 0.001, Partial *η*^2^ = 0.393). In other words, the time it took to evaluate a moral, socio-conventional, and neutral scenario all statistically differed from one another. 

### 3.2. Acceptability of Transgressive and Neutral Actions Across Age 

Finally, we analyzed how age and the type of scenario predicted the acceptability of transgressions in a 3 within-subject (moral, socio-conventional, and neutral scenarios) × 3 between-subject (children, early adolescents, and adolescents) ANOVA. Again, gender was not significantly associated with any of our predictors. Thus, it was inserted as a covariate in this model, to control for its effects. There was a single statistically significant effect, which was the main within subjects’ effect for the type of scenario, *F* (2, 166) = 564.50, *p* < 0.05, Partial *η*^2^ = 0.872. Thus, the type of scenario influenced the acceptability of the transgression across participants. Participants were most likely to accept neutral scenarios, then socio-conventional transgressions, and lastly moral transgressions. The most important distinction to note is that socio-conventional transgressions were more accepted across all age groups than moral transgressions. Repeated measures pairwise comparisons of the acceptability of scenario type across participants determined that the acceptability of the scenario differed significantly (*p* < 0.05) across all types of scenarios. Thus, across all participants, socio-conventional scenarios were significantly more acceptable than moral scenarios.

## 4. Discussion and Conclusions

The purpose of this study was twofold. First, we wanted to investigate the acceptability of transgressions and behavioral indices involved when evaluating different domain scenarios in which different types of rules (moral vs. socio-conventional rules) were broken. Specifically, we examined how statements from authority figures allowing transgressions influenced the evaluation of rule transgressions across our sample. Second, we wanted to explore the age-related differences amongst children, early adolescents, and adolescents in regard to evaluating rule transgressions in moral and socio-conventional situations, while including neutral situations as a comparison point. We hypothesized that socio-conventional transgressions would be evaluated differently than moral transgressions amongst participants, such that they would take different amounts of time to process, depending on both the type of rule scenario and the age of the participant. Furthermore, we predicted that children would take longer to process transgressions than early adolescents and adolescents.

Our findings can be summarized by exploring the reaction times and acceptability of transgressions for the scenarios across participants.

### 4.1. Behavioral Indices

Gender was not significantly associated with the time it took for participants to decipher scenarios and the acceptability of transgressions. Hence, we only controlled for the effect of gender statistically. Whereas the literature does suggest a gender difference in regard to the evaluation of moral dilemmas [20], we did not observe a gender effect. This result may indicate that neuropsychological mechanisms implicated in evaluating transgressions of different moral domains may not be susceptible to gender effects when other factors, such as age, are taken into account. Put another way, the time it takes to evaluate a rule transgression, across our sample, may be more dependent on developmental milestones measured through reaction time than gender.

Moral rule scenarios took the longest time, then socio-conventional rule scenarios, and lastly neutral scenarios. These findings were unexpected, as they contradicted the current literature [16]. It was expected that moral scenarios would take the least amount of time to evaluate, given the universality of their rules [4]. However, socio-conventional scenarios were completed in the least amount of time. These results may question the axioms of the moral domain theory, as they suggest longer reaction time for scenarios eliciting higher emotion activation (i.e., the scenarios in which moral rules were broken). Thus, the difference in reaction time may lie not in the structure of moral knowledge in separate domains, but in the emotions elicited by different types of scenarios. Alternatively, the finding that moral scenarios took the longest time to evaluate transgressive actions may be due by the complex thinking required to analyzing situations where moral rules (i.e., rules that should be independent of authorities’ statements) were broken, because of an authority’s allowance, when compared with socio-conventional scenarios that are authority rule dependent. In the experiment, children were asked to think about the rules before evaluating the permissibility of a transgression. These rules were set by the principal, a pertinent and relevant authority figure for their social world, who either allowed or denied the transgression. This may explain why socio-conventional transgressions were more quickly assessed than moral transgressions, as there was always a statement by an authority figure, allowing a rule to be broken. This clear rule we used may have made the evaluation of socio-conventional rule transgressions easier and more automatic than evaluation of moral-rule transgressions. To our knowledge, our study is one of the first to explore the socio-conventionality of transgressions. Thus, further research should investigate this phenomenon.

With regard to age, as expected, children took the longest amount of time to solve all types of scenarios, followed by early adolescents, and lastly adolescents. In addition, the analyses determined a statistically significant interaction between the type of scenario and the age of the participant when examining the reaction time of all three types of scenarios. This finding is supported by the current literature [2,21]. The amount of time it took for participants to evaluate a transgression depended on both the type of scenario they were evaluating and their age. In particular, adolescents took significantly less time to evaluate socio-conventional transgressions than the rest of participants, whereas children took the longest to evaluate moral transgressions than all other participants. In accordance with the results from the previous literature [4], this finding suggests that the ability to distinguish different domains of rules increases with age and, in comparison to younger youths, adolescents are more able to understand socio-conventionality, thus making faster decisions about socio-conventional transgressions. This trend may also indicate an increase with age of the cognitive skills implicated in moral reasoning.

### 4.2. Acceptability of Types of Transgressions

Across all age levels, transgressions of neutral scenarios were most accepted, followed by socio-conventional transgressions and lastly moral transgressions. The important distinction to note is that socio-conventional transgressions were more acceptable than moral transgressions. This finding is supported by the literature [2]. This supports the notion that transgressions of socio-conventional rules may be more acceptable, given the context-specific nature of these rules and their reliance on authority statements, which are typically based on specific contexts. Furthermore, socio-conventional rules are typically based upon an authority figure and rules pertinent to the society within which individuals find themselves, whereas moral rules are typically described as being more universal and are upheld regardless of context and situation (2008) [1].

When evaluating the acceptability of the transgression across the types of scenarios, there was only a significant main effect: The acceptability of the transgression was dependent on the type of scenario. Participants were most likely to accept neutral scenarios, then socio-conventional transgressions, and lastly moral transgressions. This finding is supported by the literature [4,21]. Moral transgressions were deemed the least acceptable. This may be due to the universality of the rules typically imbedded within moral scenarios which would be supported by the literature [7]. Similarly, socio-conventional transgressions may be more accepted, given their context-specific nature, which makes them more malleable in nature.

### 4.3. Limitations and Future Directions

There are a few limitations to note in regard to our experiment. To begin, the sample size can be larger, given the breath of these analyses. It would have been beneficial for the analyses to have a larger participant pool. Nevertheless, the precision of the assessment of the behavioral indices (reaction times) makes the analyses powerful enough. Furthermore, we conducted a post hoc power analysis with the program G*Power [22] to check whether our lack of results may be due to a lack of statistical power. We set the power (1 - β) at 0.80 and α = 0.05, two-tailed. The analysis indicated that a sample size of 54 should be enough to detect an effect of 0.20, thus confirming the overall properness of our sample size. As a second possible limitation, we did not collect longitudinal data. Our goal was to explore the age-related differences in moral development. Thus, we decided to perform a cross-sectional analysis. If we were to have focused on developmental trends, we would have employed a longitudinal design.

Future research should explore the developmental differences and behavioral indices across a longitudinal study. Furthermore, studies should explore the neurological regions implicated in evaluating moral and socio-conventional rule situations. The link should be made between the moral domain theory and the neurological regions implicated in moral decision making. Greene and colleagues [8] determined the different parts of the brain that are activated when making decisions on personal and impersonal dilemmas. However, it could be useful to determine whether there are differences in brain activation across moral and socio-conventional rule domains. The implications of an authority figure seem to determine the time it takes for all participants, regardless of age, to evaluate the acceptability of a transgression. Hence, it would be interesting to determine whether this can be attributed to the different brain areas activated in these decision-making processes. This study was conceived and realized as a preliminary study to investigate activation of the brain areas in evaluating different rule domains. The next step in the larger project will be to explore findings from this study in a fMRI experiment.

Furthermore, notwithstanding the possible limitations we listed, there were several novelties in this study and this experiment provided novel findings to contribute to the literature of moral development. To begin, our age group was diverse and allowed a broader understanding of how moral development changes across development. Hence, even if this study is not longitudinal, still it does provide an interesting perspective of change. In addition, our study focused on the socio-conventionality of a scenario. The socio-conventional scenarios were based solely on authority statements, without any nuances of moral rule scenarios within them. Furthermore, we explored the acceptability of transgressions based on only authority statements, to see whether this would influence the acceptability of a transgression. This provides a better criterion that can be used to distinguish the differences found between reaction times and acceptability of transgressions across moral and socio-conventional scenarios.

Taken together, our findings offer a unique insight into the potential different processes that are involved in making moral decisions and evaluating the acceptability of a transgression. In particular, our findings add to the sparse literature that aims to understand the behavioral indices and neuropsychological implications in moral development across youth.

## Figures and Tables

**Table 1:** Mean and standard deviations for reaction time and acceptability of transgressive/neutral actions.

		Reaction Time			Acceptability of Actions		
Age (Years)	*N*	Moral Transgressions *M (SD)*	Socio-Conventional Transgressions *M (SD)*	Neutral Actions *M (SD)*	Moral Transgressions *M (SE)*	Socio-Conventional Transgressions *M (SE)*	Neutral Actions *M (SE)*
9	24	2.70 (2.12) ^A^	1.79 (1.03) ^B^	3.72 (2.61) ^B^	0.030 (0.017)	0.223 (0.046)	0.906 (0.017)
12	32	1.84 (1.0) ^B^	1.35 (1.01) ^B^	2.31 (1.51) ^A^	0.054 (0.014)	0.288 (0.040)	0.880 (0.014)
15	31	1.21 (0.60) ^A^	0.87 (0.41) ^B^	1.48 (0.73) ^B^	0.063 (0.015)	0.354 (0.041)	0.948 (0.015)
Total	87	1.85 (1.57)	1.30 (0.92)	2.40 (1.91)	0.050 (0.08)	0.294 (0.23)	0.911 (0.087)

Note: Acceptability of transgressive/neutral actions was coded as 1, rejection as 0. Superscript A denotes the age group that significantly differs from the others. Superscript B denotes the age groups are not significantly different from each other.

**Table 2:** Associations (Pearson *r*) between reaction time and acceptability of transgressive/neutral actions with age and gender (*N* = 87).

RT/Transgression	Gender	Age	Moral Transgressions	Socio-Conventional Transgressions	Neutral Actions
Gender	−	−0.062	0.071	0.209	0.200
Age	−0.062	−	−0.375 *	−0.401 *	0.196
Moral transgressions	−0.031	0.160	−	0.782 *	0.035
Socio-conventional transgressions	0.050	0.233 *	0.661 *	−	0.061
Neutral actions	0.200	0.196	0.101	0.265 *	−

Note. RT = reaction time in seconds. Correlations above the diagonal represent the associations between reaction time, age, and gender. Correlations below the diagonal represent the associations between acceptability of actions (coded as 1, rejection as 0), age, and gender. * *p* < 0.05.
